# Effects of *p*-Cresol on Senescence, Survival, Inflammation, and Odontoblast Differentiation in Canine Dental Pulp Stem Cells

**DOI:** 10.3390/ijms21186931

**Published:** 2020-09-21

**Authors:** Mohammed Zayed, Koichiro Iohara

**Affiliations:** 1 Department of Stem Cell Biology and Regenerative Medicine, National Center for Geriatrics and Gerontology, Research Institute, Obu, Aichi 474-8511, Japan; iohara@ncgg.go.jp; 2 Department of Surgery, College of Veterinary Medicine, South Valley University, Qena 83523, Egypt

**Keywords:** dental pulp stem cells, senescence, apoptosis, aged teeth, differentiation, pulp regeneration

## Abstract

Aging, defined by a decrease in the physical and functional integrity of the tissues, leads to age-associated degenerative diseases. There is a relation between aged dental pulp and the senescence of dental pulp stem cells (DPSCs). Therefore, it is important to investigate the molecular processes underlying the senescence of DPSCs to elucidate the dental pulp aging mechanisms. *p*-Cresol (PC), a uremic toxin, is strongly related to cellular senescence. Here, age-related phenotypic changes including senescence, apoptosis, inflammation, and declining odontoblast differentiation in PC-treated canine DPSCs were investigated. Under the PC condition, cellular senescence was induced by decreased proliferation capacity and increased cell size, senescence-associated β-galactosidase (SA-β-gal) activity, and senescence markers p21, IL-1β, IL-8, and p53. Exposure to PC could stimulate inflammation by the increased expression of IL-6 and cause the distraction of the cell cycle by the increased level of Bax protein and decreased Bcl-2. The levels of odontoblast differentiation markers, dentin sialophosphoprotein (DSPP), dentin matrix protein 1, and osterix, were decreased. Consistent with those findings, the alizarin red staining, alkaline phosphatase, and DSPP protein level were decreased during the odontoblast differentiation process. Taken together, these findings indicate that PC could induce cellular senescence in DPSCs, which may demonstrate the changes in aging dental pulp.

## 1. Introduction

Promoting the health of older people can be done by conserving the function of the oral cavity [1]. The dental pulp plays an important role in maintaining longevity through involvement in the immune defense system and acting as a physical barrier. Dental pulp-derived stem cells (DPSCs) have an impressive acuity for a variety of applications in regenerative medicine [2], including osteogenic [3], angiogenic [4], neurogenic [5,6], chondrogenic [7], and skeletal muscle [8]. Moreover, their function in regenerating the dentin–pulp complex has been highlighted [9]. The neural or glial cell origin of pulp mesenchymal stem cells (MSCs) provides potential neurovascularization, multipotency, and immunomodulation [2,10,11]. The above-mentioned reports show definitively that DPSCs are a promising candidate for tissue cell-based therapy, particularly in dental translational medicine [2,10,12,13]. However, most of their physiological functions are decayed by aging pulp [14].

Several pathological changes develop in aging tissues, including cellular senescence and stem cell tiredness [15]. It has been shown that age-related changes in the dental pulp should be considered in the dentin/pulp regeneration of aged teeth [16,17]. Our understanding of dental pulp regeneration by DPSC transplantation positions it as a promising approach [12,18]. However, reduced DPSC functions in aged teeth in vitro and the inhibition of the regeneration process in vivo have been demonstrated [17,19], making the regeneration of aged pulp a challenge. There is a strong relation between aged teeth and senescent DPSCs [14]. As a result, it is essential to identify the molecular processes underlying the senescence of DPSCs and to determine a means to regulate these mechanisms to conserve healthy teeth. Previous reports have shown the induction of cellular senescence in MSCs [20,21] and DPSCs [22,23]. However, no reports have shown the effect of *p*-cresol (PC) in DPSCs.

PC, a protein binding compound, is one of the main uremic toxins that accumulate in the body tissues with advancing age [24]. Additionally, PC is markedly accumulated in the tissues of chronic kidney disease patients, inducing inflammatory and oxidative reactions [25]. It has been shown that PC inhibits the proliferation of endothelial cells and decreases endothelial wound healing [26]. Chang and colleagues demonstrated that PC induces cytotoxicity, inflammation, and cell cycle arrest in endothelial and mononuclear cells [25]. In addition, PC has been shown to cause cellular senescence and damage to the cell membrane of bone marrow-derived MSCs and impair their function [27]. PC also has been demonstrated to induce senescence in adipose tissue-derived MSCs and periodontal ligament cells [28,29] and to decrease cell differentiation [30]. Altogether, PC is assumed to induce cellular senescence and therefore tissue aging, due to its connection to inflammation and cell cycle arrest [25], which are stimulating factors of aging [15,31]. According to reports, senescence and chronic inflammation affect the properties of DPSCs for dentin-pulp regeneration [32]. As a consequence, the outcome of transplanted stem cells might be site-related rather than origin-related [33]. The serum level of PC is much higher in older than in younger individuals [34,35], suggesting a correlation between PC and tissue senescence, including dental pulp.

The role of PC in regulating the senescence of DPSCs is unknown. Here, we investigated the effect of PC in canine DPSCs, examining its effect on cellular senescence by analyzing the proliferation rate, cell size, β-galactosidase staining, and expression of senescence markers. Furthermore, the inflammation and apoptosis outcomes were investigated. Odontoblast differentiation (OD) was further evaluated within senescence-related phenotypic changes induced by PC.

## 2. Results

### 2.1. Reduced Proliferation Rate and Increased Cell Size and Senescence Induced by PC

**Figure 1:**
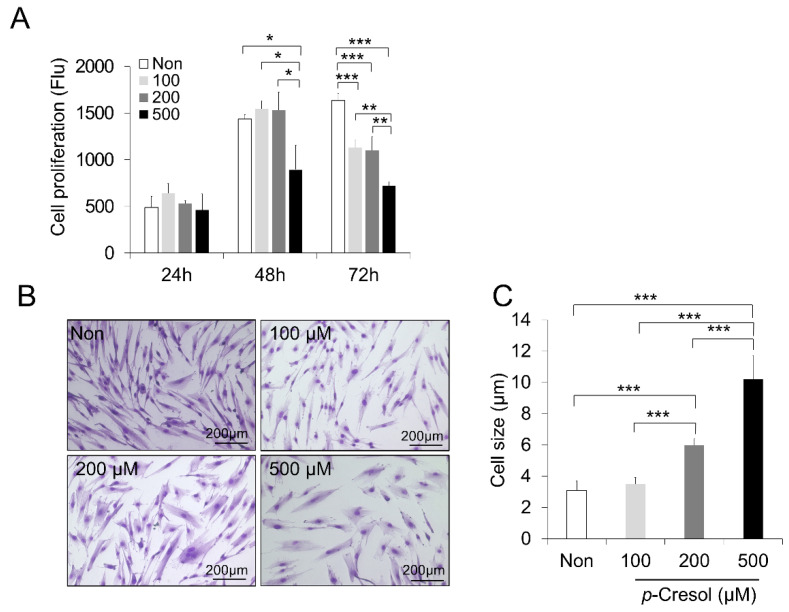
*p*-Cresol (PC) decreased the proliferation rate and increased the cell size in canine dental pulp stem cells (DPSCs). (**A**) After treatment with doses of PC (100, 200, and 500 μM) for 24, 48, and 72 h, the proliferative capacity was measured (*n* = 3). Data represent the mean ± standard deviation; * *p* < 0.05, ** *p* < 0.01, *** *p* < 0.001. (**B**) Representative images show changes associated with DPSC cell size after treatment with PC (100, 200, and 500 μM) for 72 h. Representative images are shown from one of four independent experiments. (**C**) Measurement of cell size shows a significant increase in the diameter of PC-treated DPSCs. Data represent the mean ± standard deviation; *** *p* < 0.001.

To detect the optimum concentration of PC, DPSCs were treated for 24, 48, and 72 h at three doses of 100, 200, and 500 μM. To examine the senescence effect of PC, the proliferation rate was evaluated by a PrestoBlue cell viability reagent (Figure 1A). The proliferation assay showed that 100, 200, and 500 μM decreased the proliferative capacity when compared to the non-treated control after 72 h (*p* < 0.001). The concentration of 500 μM showed a significant difference compared to 100 and 200 μM (*p* < 0.01) (Figure 1A). In addition, the PC effect on cell morphology showed that treatment at 200 and 500 μM increased the cell size significantly compared to the control and 100 μM at 72 h (*p* < 0.001). Moreover, the concentration of 500 μM showed a significant difference compared to 200 μM (*p* < 0.001) (Figure 1B,C).

**Figure 2:**
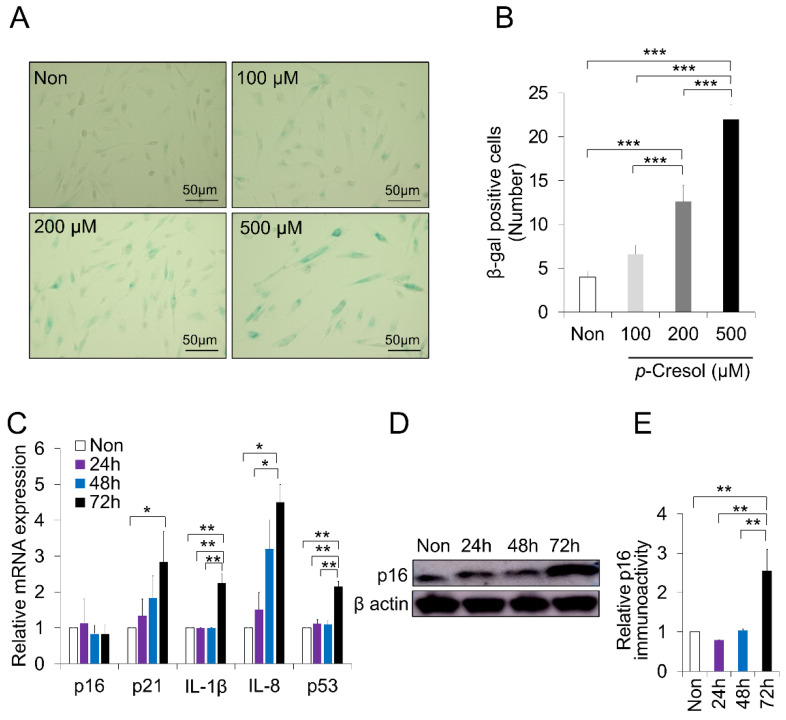
*p*-Cresol (PC) increased senescence-associated beta-galactosidase (SA-β-gal) activity and senescence markers. (**A**) Canine dental pulp stem cells (DPSCs) were subjected to PC (100, 200, and 500 μM) for 72 h. Representative images of SA-β-gal staining activity are illustrated. (**B**) Numbers of SA-β-gal-positive cells are illustrated. SA-β-gal-positive cells were significantly increased in PC-treated DPSCs with 200 and 500 μM; *** *p* < 0.001. (**C**) Gene expression of p16, p21, IL-1β, IL-8, and p53 after DPSCs were treated with PC (500 μM) for 24, 48, and 72 h by RT-PCR analysis; * *p* < 0.05, ** *p* < 0.01. (**D**) Western blot was used to analyze the protein level of p16 in PC-treated DPSCs. (**E**) Quantitative analysis of p16 immunoblot; ** *p* < 0.01. All the data are expressed as means ± standard deviations (*n* = 3).

One of the important markers in inducing cellular senescence is senescence-associated β-galactosidase (SA-β-Gal) activity [36]. To verify whether PC causes the cell senescence of DPSCs, SA-β-Gal-stained DPSCs were further evaluated. The number of DPSCs expressing the SA-β-Gal stain was significantly increased by PC (200 and 500 μM) (Figure 2A). In addition, the number of positive SA-β-Gal-stained DPSCs was significantly increased at a concentration of 500 μM compared to 200 μM (*p* < 0.001) (Figure 2B). These results indicate that PC at a concentration of 500 μM could induce cellular senescence in DPSCs.

### 2.2. Increased Senescence Markers Induced by PC

We then aimed to provide support for the senescence effect by analyzing well-known senescence-associated markers. Consistent with our expectation, PC treatment (500 μM) at 72 h significantly increased the senescence marker expression relative to the non-treated control: p21 (*p* < 0.05), IL-1β (*p* < 0.01), IL-8 (*p* < 0.05), and p53 (*p* < 0.01) (Figure 2C). In comparison, PC (500 μM) at 72 h significantly increased IL-1β (*p* < 0.01), IL-8 (*p* < 0.05), and p53 (*p* < 0.01) compared to 24 h time point. The gene expression of p16, a biological marker for cellular senescence, did not change; however, the protein expression was substantially increased (*p* < 0.01) (Figure 2D,E). These results confirm that PC (500 μM) significantly induced the senescence of DPSCs.

### 2.3. PC-Induced Apoptosis in DPSCs

**Figure 3:**
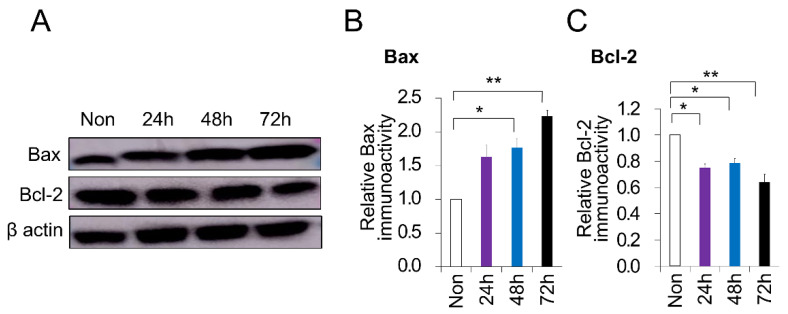
Induced apoptosis in canine dental pulp stem cells (DPSCs) treated with *p*-cresol (PC, 500 μM). (**A**) Expression of apoptosis-associated proteins analyzed by Western blot in DPSCs exposed to PC for 24, 48, and 72 h. Quantitative analysis of (**B**) Bax and (**C**) Bcl-2 immunoblot. * *p* < 0.05, ** *p* < 0.01. All the data are expressed as means ± standard deviations (*n* = 3).

To evaluate the impact of PC on the survival of DPSCs, DPSCs were stimulated with PC (500 μM) for different time points, and the protein level of Bax and Bcl-2 was further evaluated. The exposure of DPSCs to PC for 72 h induced cell apoptosis, as indicated by the increased expression of Bax, a regular apoptosis marker, and decreased Bcl-2, a cell survival marker, compared to the non-treated control (*p* < 0.01) (Figure 3A–C). Moreover, the treatment of PC after 48 h induced apoptosis by the increased expression of Bax and decreased Bcl-2 compared to the non-treated control (*p* < 0.05). No differences between the time points of treatment were demonstrated (Figure 3B,C). Taken together, these data suggest a disturbed cell cycle effect of PC on DPSCs, which validates the senescence and aging outcome.

### 2.4. p-Cresol-Induced Inflammation in DPSCs

**Figure 4:**
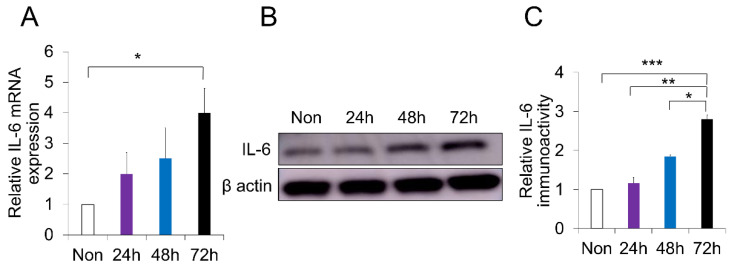
Induced inflammation in canine dental pulp stem cells (DPSCs) treated with *p*-cresol (PC, 500 μM). (**A**) Significant high expression of inflammatory marker IL-6 in PC-treated DPSCs at 72 h; * *p* < 0.05 (*n* = 3). (**B**) Immunoblot of IL-6 in PC-treated DPSCs for different time points by Western blot analysis. (**C**) Quantitative analysis of IL-6. * *p* < 0.05, ** *p* < 0.01, *** *p* < 0.01. All the data are expressed as means ± standard deviations (*n* = 3).

Our PC treatment method not only reduced DPSC proliferation and anti-apoptosis, but induced senescence-associated phenotypes as well. Nevertheless, it remains to be verified whether our treatment can explain the molecular pathways related to aging. RT-PCR analysis demonstrated that PC-induced inflammation in canine DPSCs increased the expression level of IL-6, a well-known inflammatory marker (*p* < 0.05) (Figure 4A). Furthermore, Western blot analysis showed that PC-treated DPSCs had an increased protein level of IL-6 (Figure 4B,C). There was a significant difference between PC-treated DPSCs at 72 h compared to 48 h, 24 h, and the non-treated control (*p* < 0.05, *p* < 0.01, *p* < 0.001, respectively) (Figure 4C). These data indicate that PC represented a correlated senescence and aging phenotype in DPSCs by inducing inflammation.

### 2.5. p-Cresol-Inhibited Odontoblast Differentiation in DPSCs

**Figure 5:**
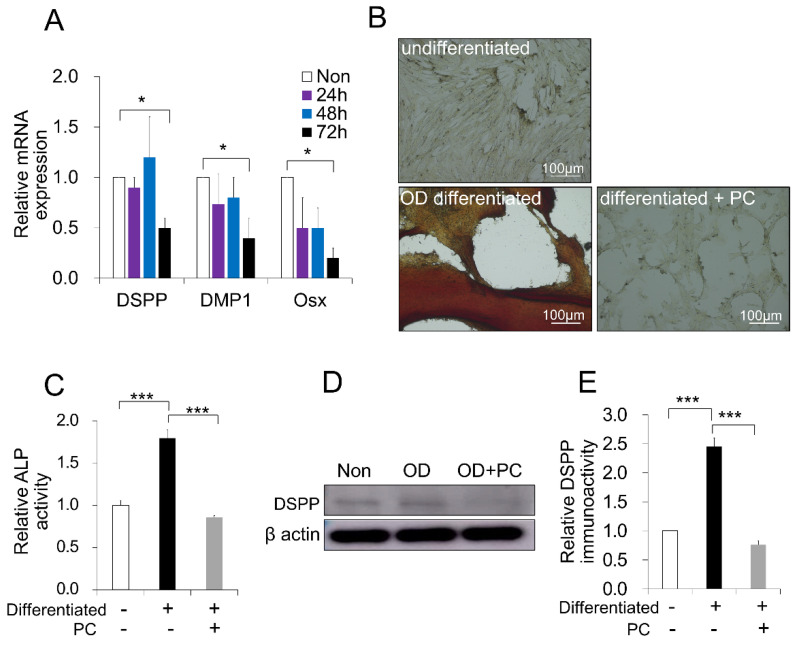
*p*-Cresol (PC, 500 μM) inhibits the differentiation potential of dental pulp stem cells (DPSCs) into odontoblasts (OD). (**A**) After treatment with PC (500 μM) at 24, 48, and 72 h, the odontogenic and mineral deposit markers were evaluated by RT-PCR (* *p* < 0.05). (**B**) Representative images of the odontoblast differentiation of DPSCs (Alizarin Red S staining) with or without PC treatment. (**C**) Alkaline phosphatase (ALP) activity in DPSCs treated with or without PC over 14 days. (*** *p* < 0.001). (**D**) DSPP expression in PC-treated DPSCs over 14 days by Western blot analysis. (**E**) Quantitative analysis of DSPP immunoblot; ** *p* < 0.01. All the data are expressed as means ± standard deviations (*n* = 3).

Aging and senescence decay the differentiation properties of MSCs. To verify whether the senescence effect of PC-treated DPSCs could be ascertained, the OD activity was examined. RT-PCR analysis demonstrated that the treatment of PC over 72 h inhibited the differentiation potential of DPSCs into odontoblasts by the decreased gene expression of dentin sialophosphoprotein (DSPP), dentin matrix protein 1 (DMP1), and osterix (Osx), markers for odontogenic and dentin mineralization, compared to the non-treated control (*p* < 0.05) (Figure 5A). There was no difference between treatments at time points of 24, 48, and 72 h. To further verify the effect of PC on OD, DPSCs were cultured in OD media with or without PC for 14 days in vitro. The quantification of odontogenesis was identified by the alizarin red staining of calcium phosphate precipitates. We found that PC revealed less mineral deposition compared to the positive control without PC (Figure 5B). Moreover, an alkaline phosphatase (ALP) assay showed that PC significantly decreased the ALP activity compared to no treatment (*p* < 0.001) (Figure 5C). Consistent with these findings, the protein level of DSPP was decreased by PC treatment (Figure 5D,E). Taken together, our PC treatment method effectively showed a wide aspect of aging at phenotypical and molecular points to validate a cellular senescence model of pulp for advanced consideration.

## 3. Discussion

Aging is one of the main processes that cause diseases such as cardiovascular disease, cancers, immune-related diseases, and neurodegenerative disorders in the older population. All tissues and organs undergo age-related changes [37], including dental pulp [14]. The regenerative potentiality of pulp tissues is decreased due to the diminishing cell density and stemness with advanced age [14]. Studies suggest that targeting age development can improve various age-related pathologies. However, aging is time-consuming, and it is difficult to achieve mechanistic experiments. There is a strong relationship between aging and senescence [38]. Thus, providing a useful cellular senescence experiment within a reasonable time frame to understand the molecular mechanisms of aging is important. Premature senescence in in vitro models by different molecules has been reported [39]. A uremic toxin, *p*-cresol, has previously been shown to be involved in aging, longevity, and age-related diseases [34]. While PC is highly implicated in the senescence of MSCs [27,28] and inflammatory diseases [25], its role in regulating the senescence and age-related phenotypic changes in DPSCs is unknown. In the current study, we showed that PC could induce senescence-related phenotypic changes in DPSCs by increasing senescence markers, apoptosis, and inflammation and inhibiting odontoblast differentiation. The conjugate of PC, *p*-cresyl sulfate, has been shown to induce a toxic effect as well [40]. A series of reactions including deamination, transamination, and decarboxylation processes are required to produce *p*-cresyl sulfate from PC [41]. In our study, none of these processes were induced. It has been shown that PC, but not *p*-cresyl sulfate, may induce the G2/M cell cycle arrest of endothelial progenitor cells [42]. Moreover, PC induced senescence in MSC-derived bone marrow and adipose tissue [27,28], inhibited the proliferation and differentiation, and increased inflammation in different cell lines [25,30]. Therefore, we think that our findings were mediated by PC itself, not its metabolites.

The stable growth arrest and acquisition of the senescence-associated secretory phenotype (SASP) are hallmarks of senescent cells [31,43]. The acquisition of SASP and permanent growth arrest lead to the stimulation of inflammation and cause tissue dysfunction [31,44,45]. For that reason, senescence is a key factor determining the quality of MSCs [46]. Studies have revealed that senescence had a harmful outcome in suppressing the proliferation and differentiation of DPSCs [47]. In addition, it has been shown that PC caused cell apoptosis and senescence in MSCs [28]. A previous report also showed that PC caused decreased cell cycle through the induction of S-phase cell cycle arrest [25]. Consistent with these findings, our PC treatment system induced DPSC senescence phenotypes through a decreased proliferation rate and enlarged the cell morphology, suggesting that PC affects the normal cell cycle. To verify that finding, the effect of PC on senescence-associated markers and SA-β-gal-positive cells was evaluated. The results showed that PC increased the senescence-related markers p21, IL-1β, and IL-8, and SA-β-gal-positive cells, a biomarker for cellular senescence, which is in agreement with an earlier study [28]. Studies confirmed that p53 plays a critical role in senescence and aging [48], and suppresses osteogenesis [49] and odontoblast differentiation [50]. In the current study, PC-treated DPSCs could upregulate the expression of p53, suggesting its role in inhibiting the odontoblast differentiation of DPSCs. p16 plays an important role in inhibiting cyclin-dependent kinases and establishing senescence-like growth arrest [51]. However, in the present study, PC-treated DPSCs did not show a difference in p16 gene expression, therefore we examined the protein level of p16, demonstrating an increased level. This contrary expression is probably due to the various complex post-transcriptional mechanisms implicated in turning mRNA into a protein [52]. On the other hand, the isolated MSCs from elderly people demonstrated the same parameters observed in senescent MSCs, such as an inferior proliferation rate, flat enlarged morphology, and positive SA-β-gal staining [53]. Consequently, our replicative senescence system may provide a proper candidate model to analysis the molecular mechanisms that direct the process of tooth aging.

The secreted SASP from senescent and aged cells is assumed to take part in constant chronic inflammation (inflamm-aging). Interleukin-6, an inflamm-aging marker, is a specific SASP component that boosts senescence [54]. PC has been reported to induce oxidation and inflammation in endothelial and mononuclear cells [25]. Our findings show that the gene and protein levels of IL-6 were increased in PC-treated DPSCs, indicating that their contribution to chronic inflammation and tissue damage [55] leads to a decline in their regenerative capacity. Moreover, damaged DNA produced from the nuclei of senescent cells may stimulate inflammatory pathways and finally lead to apoptosis. Our model shows that apoptosis-associated proteins are significantly expressed in PC-treated DPSCs, which is consistent with an earlier report [56].

The differentiation of DPSCs into odontoblasts plays an important role in dentin-pulp complex regeneration [57,58]. The dentinal extracellular matrix is built by a specialized odontoblast cell. Odontoblasts also play a defensive role in the pulp against exogenous stimuli or dental materials [59,60]. However, recent reports showed that age-related dysfunctions occurred in DPSCs [47]. Moreover, aging alters the ability of DPSCs to promote mineralization processes, and their odontogenic differentiation markers were reduced when cultured in odontogenic medium [61]. A previous report showed that PC decreased cell differentiation, and that the number of mature adipocytes was decreased [30]. In our study, PC produced a low mineral deposition activity, evidenced by alizarin red staining, and decreased odontoblast differentiation markers. Consistent with these findings, the DSPP protein level and alkaline phosphatase activity were decreased, as expected. These data suggest that the previous age-dependent decrease in pulp regeneration might have been partially due to the above-mentioned effects [19].

Considering these changes in PC-treated DPSCs, our PC treatment protocol showed the basics of senescence phenotypes and, consequently, may be of benefit to understand the molecular mechanism of aged dental pulp. However, the detailed mechanism underlying this senescence system is not yet fully understood. Further studies to examine the detailed molecular mechanisms, which will be critical for future therapeutic approaches, are still necessary. Moreover, effective DPSCs-based therapy in aged patients should be enhanced; we recently used CCR3 antagonist to retreat the adverse effects of PC on periodontal ligament cells, resulting in enhanced pulp regeneration in aged dogs [29]. In addition, other substances such as fucoidan, pioglitazone, and melatonin showed protective effects against PC-induced cellular senescence in MSCs [28,56,62]. Taken together, substances and molecules that can restore regenerative DPSCs and reserve their physiology against PC-induced senescence should be used in aged individuals, especially those with uremia.

## 4. Materials and Methods

### 4.1. Cell Culture

The study was approved by the Animal Care and Use Committee of the National Center for Geriatrics and Gerontology Research Institute and Aichi Medical University (permission #2016-5, 2017-25, issued on 25 March 2017). DPSCs were isolated according to a previous study [19] under relevant guidelines and regulations. Briefly, after extracting the upper canine teeth from 8- to 10-month-old beagle dogs (*n* = 3) (Kitayama Lab, Ina, Japan), DPSCs were isolated by enzymatic digestion and seeded on 35 mm dishes (Falcon, Corning, Tewksbury, MA, USA) in Dulbecco’s Modified Eagle’s Medium (DMEM) (Sigma-Aldrich, St. Louis, MO, USA) supplemented with 10% fetal bovine serum (FBS; GE Healthcare UK Ltd., Amersham, UK). Cultured DPSCs (4th to 6th passage) at 70% confluence were subjected to various doses of PC (100, 200, 500 μM) (Sigma-Aldrich, St. Louis, MO, USA) for the time points of 24, 48, and 72 h.

### 4.2. Determination of Proliferation and Cell Size

PrestoBlue reagent (Life Technologies, Carlsbad, CA, USA) was used to investigate the effect of PC on the DPSC proliferation and viability. DPSCs (1 × 10^3^) were cultured in 96-well plates in regular culture medium and were exposed to PC for 24, 48, and 72 h. At each time point, the culture medium was replaced by fresh medium and incubated at 37 °C for 30 min with 5% CO_2_. The PrestoBlue cell viability reagent was then applied to each well, followed by further incubation for 2 h. Fluorescence was measured using a SpectraMax Gemini XPS/EM Plate Reader (Molecular Devices, San Jose, CA, USA) at excitement 535 and emission 615.

Phase-contrast microscopy (KEYENCE, Osaka, Japan) was used to evaluate the morphological changes in DPSCs. Briefly, the cells were fixed in methanol for 5 min. Upon the removal of methanol, the cells were stained by Giemsa reagent (Sigma-Aldrich, St. Louis, MO, USA). The ImageJ software (version 1.52, imagej.nih.gov) was used to measure the average cell size of a total of 4 field images for 3 separate wells.

### 4.3. β-Galactosidase Staining

The SA-β-Gal activity was measured using a β-gal staining kit (Cell Signaling Technology, Danvers, MA, USA) at pH 6 based on the manufacturer’s instructions. Following overnight incubation at 37 °C (without CO_2_), senescent cells were known by their blue staining under a phase-contrast microscope. The stained (blue, positive) cells were counted in 5 independent cultures. The experiment was performed in triplicate.

### 4.4. Quantitative Reverse Transcription Real-Time PCR

Upon the treatment of canine DPSCs (5 × 10^5^ cells in a 6 cm^2^ culture dish) with PC (500 μM) for 24, 48, and 72 h, TRIzol (Thermo Fisher Scientific, Waltham, MA, USA) was used to isolate RNA. Reverse transcription reactions of RNA were carried out by the ReverTra Ace-α kit (Toyobo, Japan). The mRNA levels were measured for the canine senescence markers p16, p21, IL-1β, IL-8, and p53; inflammatory marker IL-6; and odontogenic and mineralized markers DSPP, DMP1, and Osx (Table 1). RT-PCR analysis of RNAs was conducted using a 7500 real-time PCR system (Applied Biosystems, Foster City, CA, USA). The threshold cycle number was calculated by the ABI 7500 software.

### 4.5. Western Blot Analysis

PC-treated DPSCs under different conditions were harvested using RIPA lysis buffer (Thermo Fisher Scientific, Waltham, MA, USA) to obtain whole protein lysate. The gel electrophoresis (12% gradient gel) of protein extracts was followed by a transfer to polyvinylidene difluoride membranes using a Trans-Blot Turbo system (BioRad, Hercules, CA, USA). Blots were blocked with 5% skim milk in Phosphate Buffered Saline with Tween 20 buffer, and incubated overnight with the following proteins: p16 (1:1000; BD Biosciences, San Jose, CA, USA), Bax (1:250; BD Biosciences), Bcl-2 (1:500; BD Biosciences), IL-6 (1:500; Abcam, Cambridge, MA, USA), DSPP (1:500; Bioss, Boston, MA, USA), and β-actin (1:1000; Cell Signaling Technology). Membranes were then incubated with either horseradish peroxidase (HRP)-conjugated anti-rabbit (1:5000) or anti-mouse (1:1000) secondary antibodies (Cell Signaling Technology). Following development, the blots were imaged and analyzed using the ImageJ software.

### 4.6. Odontoblast Differentiation and Alkaline Phosphatase Assay

Canine DPSCs (3 × 10^5^ cells) were seeded in 6-well cell culture plates (Falcon, Corning, Tewksbury, MA, USA), at 70% confluence, then were cultured in an odontoblast differentiation medium containing L-ascorbic acid-2-phosphate (50 μg/mL; Sigma-Aldrich, St. Louis, MO, USA), β-glycerophosphate (10 mM; Sigma-Aldrich), and dexamethasone (100 nM; Sigma-Aldrich) with or without PC (500 μM) for 14 days. Mineral nodules were stained with alizarin red stain (Wako Pure Chemical Industries, Japan) after 4% paraformaldehyde fixation for 10 min at room temperature. To further assess the effect of PC on odontoblast differentiation, the protein level of DSPP was determined by Western blot analysis, as previously mentioned. For the ALP test, cell lysates were extracted after odontoblast differentiation, and the ALP activity was assessed and analyzed as directed by the supplier (LabAssay ALP; Wako Pure Chemical Corporation, Osaka, Japan).

### 4.7. Statistical Analyses

Statistical analysis was carried out using SPSS (IBM, Armonk, NY, USA). Statistical differences were determined using either a Student’s *t*-test or an analysis of variance (ANOVA), with Tukey’s comparison test as a post test. *p*-value < 0.05 was considered statistically significant.

## 5. Conclusions

In conclusion, PC suppressed cell growth; induced senescence, apoptosis, and inflammation; and inhibited odontoblast differentiation in DPSCs. These findings may help in analyzing the mechanisms and demonstrating the characteristics of aged dental pulp and explain the age-dependent decrease in pulp regeneration by DPSCs. Taken together, our results demonstrate that PC may play an important role in DPSC senescence in association with the induction of some SASP factors.

## Figures and Tables

**Table 1:** Canine primer sequences used in real-time polymerase chain reaction analysis.

Primer Name		Primer Sequence	Size
p16	Forward	CGGAAGGTCACGCAGACAGC	124 bp
	Reverse	GCAGGGAAGAGTTGGGTTGGGT	
p21	Forward	ACCTCTCAGGGCCGAAAAC	89 bp
	Reverse	TAGGGCTTCCTCTTGGAGAA	
IL-1β	Forward	CAAGAGTCTGAGGCATTTC	214 bp
	Reverse	GGTATTTGTGGCTTATGTCC	
IL-8	Forward	ACACTCCACACCTTCCAT	143 bp
	Reverse	CTTTTGTACCCATTTTTCC	
p53	Forward	CGCAAAAGAAGAAGCCACTA	118 bp
	Reverse	TCCACTCTGGGCATCCTT	
IL-6	Forward	TCCAGAACAACTATGAGGGTGA	100 bp
	Reverse	TCCTGATTCTTTACCTTGCTCTT	
DSPP	Forward	GTCCTAGTGGGAATGGAGCA	190 bp
	Reverse	TCTTCAGGGCCATCATCTTC	
DMP1	Forward	GATAGTGCCCAAGATACCAC	120 bp
	Reverse	TCCTACCCAGTGTTCCTTAC	
Osx	Forward	ACCAATGGGCTCCTCTCAC	162 bp
	Reverse	CACTGGGCAGGCAGTCAGGA	
β-actin	Forward	AAGTACCCCATTGAGCACGG	257 bp
	Reverse	ATCACGATGCCAGTGGTGCG	

## Abbreviation

ALP	Alkaline phosphatase
Bax	BCL2 associated X
Bcl-2	B-cell lymphoma 2
DMEM	Dulbecco’s modified eagle’s medium
DMP1	Dentin matrix protein 1
DPSCs	Dental pulp stem cells
DSPP	Dentin sialophosphoprotein
FBS	Fetal bovine serum
MSCs	Mesenchymal stem cells
Osx	Osterix
PC	*p*-Cresol
RT-PCR	Reverse transcription polymerase chain reaction
SA-β-Gal	Senescence-associated beta-galactosidase
